# Assessing the potential role of ChatGPT in spine surgery research

**DOI:** 10.1002/jeo2.12057

**Published:** 2024-06-13

**Authors:** Isabel Herzog, Dhruv Mendiratta, Ashok Para, Ari Berg, Neil Kaushal, Michael Vives

**Affiliations:** ^1^ Rutgers New Jersey Medical School Newark New Jersey USA

**Keywords:** artificial intelligence, ChatGPT, machine learning, systematic reviews

## Abstract

**Purpose:**

Since its release in November 2022, Chat Generative Pre‐Trained Transformer 3.5 (ChatGPT), a complex machine learning model, has garnered more than 100 million users worldwide. The aim of this study is to determine how well ChatGPT can generate novel systematic review ideas on topics within spine surgery.

**Methods:**

ChatGPT was instructed to give ten novel systematic review ideas for five popular topics in spine surgery literature: microdiscectomy, laminectomy, spinal fusion, kyphoplasty and disc replacement. A comprehensive literature search was conducted in PubMed, CINAHL, EMBASE and Cochrane. The number of nonsystematic review articles and number of systematic review papers that had been published on each ChatGPT‐generated idea were recorded.

**Results:**

Overall, ChatGPT had a 68% accuracy rate in creating novel systematic review ideas. More specifically, the accuracy rates were 80%, 80%, 40%, 70% and 70% for microdiscectomy, laminectomy, spinal fusion, kyphoplasty and disc replacement, respectively. However, there was a 32% rate of ChatGPT generating ideas for which there were 0 nonsystematic review articles published. There was a 71.4%, 50%, 22.2%, 50%, 62.5% and 51.2% success rate of generating novel systematic review ideas, for which there were also nonsystematic reviews published, for microdiscectomy, laminectomy, spinal fusion, kyphoplasty, disc replacement and overall, respectively.

**Conclusions:**

ChatGPT generated novel systematic review ideas at an overall rate of 68%. ChatGPT can help identify knowledge gaps in spine research that warrant further investigation, when used under supervision of an experienced spine specialist. This technology can be erroneous and lacks intrinsic logic; so, it should never be used in isolation.

**Level of Evidence:**

Not applicable.

**Abbreviations**
AI: artificial intelligence
ChatGPT: Chat Generative Pre‐Trained Transformer
Non‐SR: nonsystematic review
SR: systematic review

## BACKGROUND

Artificial intelligence (AI) has emerged as a promising tool in the field of medicine due to its ability to analyze large datasets, identify patterns and generate predictions with clinical relevance. The application of AI has been previously described in the field of spine surgery [5, 9, 18, 21, 29, 35, 39]. For instance, unique algorithms can generate predictions on outcomes of conservative versus surgical treatment in patients with spine pathology [21]. AI refers to the study of algorithms that provide machines with reasoning and cognitive abilities, such as decision‐making and problem‐solving [24]. Machine learning refers to the ability of machines to learn through pattern recognition; it is particularly suited to predictions based on existing data [10]. Machine learning has numerous applications in medicine, such as prediction of surgical site infections by creating models that encompass diagnoses, treatments and laboratory values [44]. A study conducted in 2017 found that machine learning could predict patient lung cancer staging in the Surveillance, Epidemiology and End Results Cancer Registry with high sensitivity, specificity and accuracy [6].

On November 30, 2022, Chat Generative Pre‐trained Transformer 3.5 (ChatGPT) OpenAI, created by a San Francisco‐based AI laboratory, became available to the general public [4]. Since its release, ChatGPT has garnered attention for its impressive performance on all three components of the United States Medical Licensing Exam and ability to deceive scientists with its abstract‐writing skills [15, 30]. Studies in the field of plastic surgery have explored the potential utility of ChatGPT in research generation, grant writing and patient consultations [20, 38, 49]. A study by He et al. described the possible roles of ChatGPT in spinal surgical practice, such as surgical planning, patient data collection and postoperative rehabilitation guidance [25]. ChatGPT is trained on more than 1.6 billion parameters, allowing it to interact with users, appropriately respond to questions, learn from and acknowledge previous mistakes and manipulate data [7, 41].

Research is a crucial component of the growing field of spine surgery, and thus, systematic reviews offer considerable utility. Systematic reviews are investigations that consolidate and analyze relevant findings of all available studies concerning a specific research question. Systematic reviews coincide with the practice of evidence‐based medicine: treatment of patients through the integration of personal clinical experience with findings from intensive, high‐standard research [13, 19]. Physicians utilize systematic reviews to learn about updates in the field and appropriately adjust their clinical practices. Systematic reviews can also serve as justification for further research from the perspective of granting agencies, clinical practice guideline developers and healthcare agencies [19]. As such, subject areas within the field of spine surgery that lack systematic reviews represent potential knowledge gaps. However, systematic reviews can prove to be time and resource‐intensive task, costly and time‐consuming—requiring an average of 6 months to several years to complete [47]. Furthermore, it can be difficult to ensure that a systematic review idea is both novel and clinically relevant by a researcher alone. Machine learning might have a role in ameliorating these practical concerns for spine surgeons conducting research. Therefore, the purpose of this study is to investigate the ability of ChatGPT to provide novel systematic review proposals on topics within spine surgery.

## METHODS

On 4 February 2023, ChatGPT‐3.5 was instructed to generate ten novel systematic review proposals for five popular topics in spine surgery literature: microdiscectomy, laminectomy, spinal fusion, kyphoplasty and disc replacement. This was accomplished using the instruction, ‘Give 10 novel systematic review ideas on [topic]’ for each of the five aforementioned topics. Therefore, a total of 50 systematic review ideas were generated for this study. A new ChatGPT account was utilized in order to eliminate user history bias. Furthermore, prompts for each of the five topics were given in separate chats.

For each of the 50 ChatGPT‐generated novel systematic review ideas, a comprehensive literature search was conducted. PubMed, CINAHL, EMBASE and Cochrane were separately queried for articles that covered the respective ChatGPT‐generated topic. Duplicates were removed, and all search results were assessed to verify their relevance to the ChatGPT‐generated idea. The number of nonsystematic review articles and number of systematic review papers that had been published in 2023 or prior were recorded and totalled for each generated idea. An idea was considered novel if there were no systematic reviews already published on the topic. Papers were reviewed using the full‐text article when available. If there were no published nonsystematic review articles on an idea, this was also noted. The title, lead author and date of publication were recorded for each published systematic review. If an article was published after September 2021, this was recorded. To decrease the possibility of erroneously including or removing studies, two reviewers (I.H., D.M.) separately assessed each article. If there were any discrepancies, a third reviewer (M.V.) was consulted to make the final decision. The overall accuracy rate for novelty was calculated by dividing the number of novel ChatGPT‐generated ideas by 50. Topic‐specific accuracy rates for novelty were calculated in a similar fashion for microdiscectomy, laminectomy, spinal fusion, kyphoplasty and disc replacement by dividing the number of novel ChatGPT‐generated ideas by 10.

## RESULTS

Overall, we determined that ChatGPT was able to generate 34 novel systematic review proposals, corresponding to a 68% accuracy rate. Tables 1, 2, 3, 4, 5 present the ChatGPT‐generated idea with the corresponding number of nonsystematic reviews (non‐SRs), number of SRs and novelty status for microdiscectomy, laminectomy, spinal fusion, kyphoplasty and disc replacement that were respectively identified by traditional manual literature search methods.

**Table 1:** ChatGPT‐generated systematic review ideas and respective accuracy rates for microdiscectomy: Number of nonsystematic reviews (non‐SRs), number of SRs and novelty status shown.

Ideas	Number of non‐SRs published	Number of SRs published	Novel idea? (Y/N)	Accuracy rate for novelty
Microdiscectomy				80%
Comparison of microdiscectomy outcomes between elderly and younger patients.	0	0	Y	
A systematic review of the incidence of recurrent herniation after microdiscectomy.	5	0	Y	
The impact of patient factors such as obesity and smoking on microdiscectomy outcomes.	7	0	Y	
A review of the effectiveness of different surgical approaches for lumbar microdiscectomy.	7	2	N	
The impact of preoperative imaging studies on the success of microdiscectomy.	0	0	Y	
A systematic review of the success rates and complication rates of minimally invasive microdiscectomy compared to traditional open microdiscectomy.	1	0	Y	
The influence of patient expectations on outcomes after microdiscectomy.	0	0	Y	
A systematic review of the cost‐effectiveness of microdiscectomy compared to alternative treatments for herniated lumbar discs.	3	0	Y	
The role of physical therapy and rehabilitation in improving outcomes after microdiscectomy.	8	1	N	
A review of patient‐reported outcomes after microdiscectomy, including quality of life and return to work.	6	0	Y	

**Table 2:** ChatGPT‐generated systematic review ideas and respective accuracy rates for laminectomy: Number of nonsystematic reviews (non‐SRs), number of SRs and novelty status shown.

Ideas	Number of non‐SRs published	Number of SRs published	Novel idea? (Y/N)	Accuracy rate for novelty
Laminectomy				80%
Comparison of outcomes between minimally invasive and traditional open laminectomy for spinal stenosis.	8	3	N	
Analysis of the effect of patient age and comorbidities on outcomes following laminectomy for spinal stenosis.	0	0	Y	
Assessment of the long‐term outcomes of laminectomy for degenerative disc disease.	0	0	Y	
Review of the use of nerve monitoring techniques during laminectomy to minimize the risk of nerve injury.	0	0	Y	
Analysis of the role of postoperative physical therapy in optimizing recovery following laminectomy.	2	1	N	
Comparison of laminectomy with other surgical options for the treatment of herniated discs.	10	2	Y	
Assessment of the impact of different surgical approaches on patient satisfaction after laminectomy.	0	0	Y	
Analysis of the risk of reoperation following laminectomy for spinal stenosis.	0	0	Y	
Comparison of outcomes between laminectomy and laminoplasty for the treatment of spinal stenosis.	3	0	Y	
Examination of the impact of adjuvant treatments such as epidural injections and nerve blocks on outcomes following laminectomy.	0	0	Y	

**Table 3:** ChatGPT‐generated systematic review ideas and respective accuracy rates for spinal fusion: Number of nonsystematic reviews (non‐SRs), number of SRs and novelty status shown.

Ideas	Number of non‐SRs published	Number of SRs published	Novel idea? (Y/N)	Accuracy rate for novelty
Spinal fusion				40%
The long‐term outcomes of cervical spinal fusion for the treatment of degenerative disc disease.	>100	0	Y	
The impact of smoking on spinal fusion outcomes.	>100	2	N	
The comparative efficacy of minimally invasive vs traditional spinal fusion for lumbar degenerative conditions.	3	0	Y	
The safety and effectiveness of allograft vs autograft for spinal fusion.	5	2	N	
The role of biologics in promoting spinal fusion success.	>100	12	N	
The impact of patient characteristics (age, obesity, comorbidities) on spinal fusion outcomes.	>100	5	N	
The effectiveness of spinal fusion in the treatment of spinal tumours.	0	0	Y	
The impact of revision surgery on the success of spinal fusion.	>100	0	Y	
The cost‐effectiveness of spinal fusion compared to non‐surgical treatments for chronic back pain.	10	1	N	
The effectiveness of different spinal fusion techniques for the treatment of lumbar spinal stenosis.	>100	4	N	

**Table 4:** ChatGPT‐generated systematic review ideas and respective accuracy rates for kyphoplasty: Number of nonsystematic reviews (non‐SRs), number of SRs and novelty status shown.

Ideas	Number of non‐SRs published	Number of SRs published	Novel idea? (Y/N)	Accuracy rate for novelty
Kyphoplasty				70%
Comparison of pain and functional outcomes after kyphoplasty vs vertebroplasty.	35	3	N	
Kyphoplasty outcomes in patients with osteoporotic vertebral fractures versus those with traumatic fractures.	0	0	Y	
Cost‐effectiveness of kyphoplasty versus alternative surgical and non‐surgical treatments for vertebral fractures.	8	4	N	
Long‐term safety and effectiveness of kyphoplasty in elderly patients.	6	0	Y	
The impact of patient comorbidities on kyphoplasty outcomes.	60	0	Y	
Radiological assessment of vertebral height restoration and kyphotic angle correction after kyphoplasty.	0	0	Y	
Clinical and radiological outcomes of single‐ vs multi‐level kyphoplasty.	0	0	Y	
Adjunctive therapies for improvement of kyphoplasty outcomes, such as physical therapy or pharmacotherapy.	0	0	Y	
Kyphoplasty for vertebral compression fractures in patients with spinal tumours.	>100	4	N	
Kyphoplasty in the management of vertebral fractures in patients with Ankylosing Spondylitis.	3	0	Y	

**Table 5:** ChatGPT‐generated systematic review ideas and respective accuracy rates for disc replacement: Number of nonsystematic reviews (non‐SRs), number of SRs and novelty status shown.

Ideas	Number of non‐SRs published	Number of SRs published	Novel idea? (Y/N)	Accuracy rate for novelty
Disc replacement				70%
Clinical outcomes of total disc replacement in the cervical spine.	>100	8	N	
A comparison of total disc replacement with fusion surgery in the lumbar spine.	3	0	Y	
Long‐term durability of total disc replacement in the lumbar and cervical spine.	0	0	Y	
Economic evaluation of total disc replacement compared to fusion surgery.	9	0	Y	
Patient satisfaction following total disc replacement surgery	0	0	Y	
Revision and reoperation rates following total disc replacement surgery.	22	0	Y	
A systematic review of the radiological outcomes of total disc replacement surgery.	>100	1	N	
The impact of age, gender, and body mass index on the outcomes of total disc replacement surgery.	4	0	Y	
Clinical outcomes of total disc replacement in patients with degenerative disc disease.	>100	13	N	
Total disc replacement for the treatment of herniated nucleus pulposus and radiculopathy.	3	0	Y	

Topics related to microdiscectomy and laminectomy had the highest accuracy rates—80%. Spinal fusion had the lowest accuracy rate, calculated to be 40%. Kyphoplasty and disc replacement both had accuracy rates of 70%. In addition, there was an overall 32% rate of ChatGPT generating ideas for which there were 0 nonsystematic review articles published. With this as consideration, there was a 71.4%, 50%, 33.3%, 50%, 62.5% and 51.2% success rate of generating novel systematic review ideas, for which there were also nonsystematic reviews published, for microdiscectomy, laminectomy, spinal fusion, kyphoplasty, disc replacement and overall, respectively.

## DISCUSSION

This study demonstrates that ChatGPT is a feasible tool for aiding spine surgeons in the identification of knowledge gaps and construction of novel systematic review ideas. Previous studies have shown that clinician‐machine interaction can enhance decision‐making [24]. Machine learning technology, such as ChatGPT, can serve as powerful tools for uncovering subtle or overlooked patterns in available data. ChatGPT can swiftly browse through large amounts of data, such as patient records, scientific papers and textbooks, to create novel hypotheses in medicine [14].

During the manual novelty analysis for the 50 ChatGPT‐generated ideas, 78 individual systemic reviews that were already published on those topics were identified (Appendix A). A notable limitation of ChatGPT is that it was trained on a data set last updated in September 2021 [45]. This was evident in our study, as some manually identified systematic reviews were published after this date. Specifically, eight systematic reviews were published after 2021 and were most likely not incorporated into ChatGPT training [8, 16, 17, 22, 32, 33, 42, 46]. Therefore, ChatGPT cannot provide users with completely current, accurate information more recent than its last update. Second, 11 published systematic review titles and abstracts did not include the phrases ‘systematic review’, ‘Cochrane review’ or ‘meta‐analysis’, which could have led to ChatGPT missing these articles [11, 12, 23, 26, 27, 28, 31, 36, 37, 40, 43]. Similarly, ChatGPT appeared to miss some articles that did not directly include the procedure name in the title. For instance, during the microdiscectomy portion of the study, two titles with the phrase ‘operative approaches for lumbar disc herniation’—without including ‘microdiscectomy’—failed to be acknowledged by ChatGPT [3, 48]. In these instances, the failure of ChatGPT to recognize these studies may be its lack of access to the full text of these studies since such access may require individual or institutional subscription. Finally, ChatGPT might have had issues recognizing synonyms for medical and procedural terminology or may not have yet ‘learned’ how certain terms are grouped into larger categories. For example, ChatGPT proposed the systematic review ‘The role of biologics in promoting spinal fusion success’ as novel. Manual search, however, identified multiple systematic reviews investigating the effectiveness of bone morphogenetic proteins, autologous stem cells and osteoinductive bone graft substitutes [1, 2, 34, 50]. Upon review, these studies did not have the term ‘biologics’ in their publicly available title, abstract, MeSH terms or keywords; so ChatGPT may not have been able to identify them as fitting into this larger category.

There were 16 instances of ChatGPT generating a systematic review idea for which there were no nonsystematic reviews published. This occurrence was most notable for laminectomy (50%), followed by kyphoplasty (40%), microdiscectomy (30%), disc replacement (20%) and spinal fusion (10%). To account for this discrepancy, this study also calculated the overall accuracy rate for which there were nonsystematic reviews published on that topic. In these cases, the absence of nonsystematic reviews was generally indicative of a paucity of clinical studies on the proposed topic. Given this, these 16 systematic review proposals could not currently be performed as actual studies, but the topics generated may shed light on research gaps in spine surgery. For instance, these ChatGPT‐generated ideas could inspire other types of studies within spine surgery, such as cohort studies, randomized controlled trials and so on. The potential role of ChatGPT in generating salient, novel ideas for other study types should be explored in future studies.

ChatGPT has been observed to sometimes provide answers that appear plausible at first glance but are actually meaningless or illogical. To a degree, this is inevitable, as the current ChatGPT model lacks common sense and practical knowledge. For example, in the present study, it is unclear why ChatGPT missed several relevant systematic reviews for kyphoplasty and disc replacement since the titles of these articles closely resembled the respective ChatGPT‐generated idea. Despite these shortcomings, ChatGPT can still facilitate improvement in spine surgery research, which, in turn, serves to improve patient management and outcomes through the practice of evidence‐based medicine. Therefore, we recommend that ChatGPT should never be used blindly but utilized in conjunction with the expertise of a spine surgeon to identify and refine research ideas. We also do not recommend utilizing ChatGPT or other large language models for authorship of research manuscripts, as such would compromise scholarly integrity.

This was a pilot study that aimed to determine whether ChatGPT could generate suggestions for both systematic reviews and nonsystematic reviews in a novel fashion. The rationale was that if there were nonsystematic reviews on a topic, it would suggest that there was potential feasibility for designing a systematic review on that topic. For the topics generated that had no systematic reviews or nonsystematic reviews, it was believed these would be topics that merit individual attention by a research team to determine their feasibility. Narrowing the eligibility criteria further may have discarded ideas with potential due to nuances of wording in the title or content of the text at the level of an individual study. However, this is certainly an important point, and determining if ChatGPT can independently establish feasibility should be evaluated in future studies. This study found several topic ideas to be meaningless or illogical, further demonstrating that feasibility warrants evaluation. Tables 1, 2, 3, 4, 5 list each ChatGPT topic suggestion verbatim so that readers can adequately comprehend the nature of these ideas.

This study was intended as proof of concept and should be expanded in future studies. First, this study only instructed ChatGPT to generate 50 ideas; increasing the number of generated ideas might affect the accuracy rate. Investigating the effects of specificity in regard to topics might also improve the accuracy rate, as microdiscectomy, laminectomy, spinal fusion, kyphoplasty and disc replacement are relatively broad terms within the field. This study did not fully take advantage of the supervised and reinforcement learning nature of ChatGPT. Future studies should experiment with this, as this is expected to increase accuracy rates and general convenience. A limitation of this study is that the phrasing of prompts given to ChatGPT could have an impact on the results; this study does not investigate whether different prompts significantly alter the generated response. Prompt phrasing was kept the same for each of the five topics: microdiscectomy, laminectomy, spinal fusion, kyphoplasty and disc replacement. To increase standardization between LLM studies, the phrasing for prompts in this study was modelled from similar literature [20]. The purpose of this preliminary study was simply to assess ChatGPT's ability to generate novel systematic ideas without additional guidance or corrections.

Due to the complexity of surgical interventions, technologies and approaches utilized to manage spine‐related disorders, systematic reviews play an important role in the field of spine surgery. Systematic reviews can bridge the gaps in knowledge of a plethora of spine topics, but the sheer volume of data can be overwhelming for researchers to manually identify and analyze. Machine learning technologies, such as ChatGPT, may solve this problem: allowing researchers to analyze large volumes of published literature more quickly while maintaining acceptable accuracy.

## CONCLUSION

ChatGPT, an AI chatbot, successfully generated novel systematic review ideas related to spine surgery at an overall rate of 68%. As this technology improves through supervised learning, reinforcement learning and dataset expansion, there is a role for ChatGPT as a research tool. The application of ChatGPT can identify knowledge gaps in spine research that warrant further investigation and improve research efficiency by eliminating the need to manually examine hundreds to thousands of publications. With more specific instructions and user‐driven modulation, the accuracy of ChatGPT in this role can be increased.

## AUTHOR CONTRIBUTIONS

All authors contributed to the study conception and design. Material preparation, data collection and analysis were performed by Ms. Herzog and Mr. Mendiratta. The first draft of the manuscript was written by Ms. Herzog, and all authors commented on previous versions of the manuscript. All authors read and approved the final manuscript.

## CONFLICT OF INTEREST STATEMENT

The authors declare no conflict of interest.

## ETHICS STATEMENT

The ethics statement is not available. And there were no participants included in this study.

## Data Availability

The data sets used and/or analyzed during the current study are available from the corresponding author on reasonable request.

## APPENDIX A

**Table:**

Title	Lead author	Month and year of publication
**Microdiscectomy**		
A review of the effectiveness of different surgical approaches for lumbar microdiscectomy		
Comparison of Different Operative Approaches for Lumbar Disc Herniation: A Network Meta‐Analysis and Systematic Review	Wei	July 2021
Operative Approaches for Lumbar Disc Herniation: A Systematic Review and Multiple Treatment Meta‐Analysis of Conventional and Minimally Invasive Surgeries	Alvi	June 2018
The role of physical therapy and rehabilitation in improving outcomes after microdiscectomy.		
Rehabilitation after lumbar disc surgery	Oosterhuis	March 2014
**Laminectomy**		
Comparison of outcomes between minimally invasive and traditional open laminectomy for spinal stenosis		
Minimally Invasive Versus Open Laminectomy for Lumbar Stenosis: A Systematic Review and Meta‐Analysis	Phan	January 2016
Lumbar Spinal Stenosis Associated With Degenerative Lumbar Spondylolisthesis: A Systematic Review and Meta‐analysis of Secondary Fusion Rates Following Open vs Minimally Invasive Decompression	Scholler	March 2017
Systematic Review of Cost‐Effectiveness Analyses Comparing Open and Minimally Invasive Lumbar Spinal Surgery	Eseonu	July 2022
Analysis of the role of postoperative physical therapy in optimizing recovery following laminectomy.		
Rehabilitation after lumbar disc surgery	Oosterhuis	March 2014
**Spinal fusion**		
The impact of smoking on spinal fusion outcomes		
The Effect of Smoking on the Fusion Rate of Spinal Fusion Surgery: A Systematic Review and Meta‐Analysis	Li	October 2021
A critical analysis of the literature regarding surgical approach and outcome for adult low‐grade isthmic spondylolisthesis	Kwon	February 2005
The safety and effectiveness of allograft vs autograft for spinal fusion		
Synthetic bone graft versus autograft or allograft for spinal fusion: a systematic review	Buser	October 2016
Iliac Crest Bone Graft versus Local Autograft or Allograft for Lumbar Spinal Fusion: A Systematic Review	Tuchman	September 2016
The role of biologics in promoting spinal fusion success		
Osteoconductive bone graft extenders in posterolateral thoracolumbar spinal fusion: a systematic review	Alsaleh	July 2012
Assessment of outcome following the use of recombinant human bone morphogenetic protein‐2 for spinal fusion in the elderly population	Shweikeh	June 2016
Bone Morphogenetic Proteins in Anterior Cervical Fusion: A Systematic Review and Meta‐Analysis	Zadegan	August 2017
Use of Autologous Stem Cells in Lumbar Spinal Fusion: A Systematic Review of Current Clinical Evidence	Buser	October 2021
Clinical effectiveness and cost‐effectiveness of bone morphogenetic proteins in the non‐healing of fractures and spinal fusion: a systematic review	Garrison	August 2007
Allograft Versus Demineralized Bone Matrix in Instrumented and Noninstrumented Lumbar Fusion: A Systematic Review	Buser	June 2018
The minimally effective dose of bone morphogenetic protein in posterior lumbar interbody fusion: a systematic review and meta‐analysis	Lytle	August 2020
Effectiveness and harms of recombinant human bone morphogenetic protein‐2 in spine fusion: a systematic review and meta‐analysis	Fu	June 2013
A Systematic Review of Lumbar Fusion Rates With and Without the Use of rhBMP‐2	Galimberti	July 2015
Bone morphogenetic protein use in spine surgery‐complications and outcomes: a systematic review	Faundez	June 2016
Osteoinductive bone graft substitutes for lumbar fusion: a systematic review	Agarwal	December 2009
Complications associated with the use of the recombinant human bone morphogenetic proteins for posterior interbody fusions of the lumbar spine	Chrastil	July 2013
The impact of patient characteristics (age, obesity, comorbidities) on spinal fusion outcomes		
Clinical Outcomes of Bariatric Surgery Before Spinal Fusion: A Systematic Review	Luxenburg	February 2023
Outcome of lumbar spinal fusion surgery in obese patients: a systematic review and meta‐analysis	Lingulta	October 2015
Impact of Elevated Body Mass Index on Surgical Outcomes for Patients Undergoing Cervical Fusion Procedures: A Systematic Review and Meta‐Analysis	Zhang	February 2020
Ambulatory Lumbar Fusion: A Systematic Review of Perioperative Protocols, Patient Selection Criteria, and Outcomes	Subramanian	February 2023
Fusion versus nonoperative management for chronic low back pain: do comorbid diseases or general health factors affect outcome?	Choma	October 2011
The cost‐effectiveness of spinal fusion compared to non‐surgical treatments for chronic back pain		
Cost‐effectiveness of surgical treatment for degenerative spondylolisthesis and spinal stenosis	Harrop	October 2014
The effectiveness of different spinal fusion techniques for the treatment of lumbar spinal stenosis		
Is Indirect Decompression and Fusion More Effective than Direct Decompression and Fusion for Treating Degenerative Lumbar Spinal Stenosis With Instability? A Systematic Review and Meta‐analysis	Gagliardi	March 2023
Decompression and coflex interlaminar stabilisation compared with conventional surgical procedures for lumbar spinal stenosis: A systematic review and meta‐analysis	Li	April 2017
Posterolateral Fusion Versus Posterior Lumbar Interbody Fusion: A Systematic Review and Meta‐Analysis of Randomized Controlled Trials	Said	June 2022
Systematic Review of Cost‐Effectiveness Analyses Comparing Open and Minimally Invasive Lumbar Spinal Surgery	Eseonu	July 2022
**Kyphoplasty**		
Comparison of pain and functional outcomes after kyphoplasty vs vertebroplasty		
Outcomes of vertebroplasty compared with kyphoplasty: a systematic review and meta‐analysis	Gu	June 2016
Systematic Review and Meta‐Analysis of 3 Treatment Arms for Vertebral Compression Fractures: A Comparison of Improvement in Pain, Adjacent‐Level Fractures, and Quality of Life Between Vertebroplasty, Kyphoplasty, and Nonoperative Management	Halvachizadeh	October 2021
Comparing effects of kyphoplasty, vertebroplasty, and non‐surgical management in a systematic review of randomized and non‐randomized controlled studies	Papanastassiou	September 2012
Cost‐effectiveness of kyphoplasty versus alternative surgical and nonsurgical treatments for vertebral fractures		
Percutaneous vertebroplasty and percutaneous balloon kyphoplasty for the treatment of osteoporotic vertebral fractures: a systematic review and cost‐effectiveness analysis	Stevenson	March 2014
A Systematic Review of the Level of Evidence in Economic Evaluations of Medical Devices: The Example of Vertebroplasty and Kyphoplasty	Martelli	December 2015
Vertebroplasty and kyphoplasty—a systematic review of cement augmentation techniques for osteoporotic vertebral compression fractures compared to standard medical therapy	Robinson	May 2012
Vertebral Augmentation Involving Vertebroplasty or Kyphoplasty for Cancer‐Related Vertebral Compression Fractures: An Economic Analysis	Health Quality Ontario	May 2016
Kyphoplasty for vertebral compression fractures in patients with spinal tumors		
Vertebral Augmentation Involving Vertebroplasty or Kyphoplasty for Cancer‐Related Vertebral Compression Fractures: A Systematic Review	Health Quality Ontario	May 2016
Vertebral Augmentation Involving Vertebroplasty or Kyphoplasty for Cancer‐Related Vertebral Compression Fractures: An Economic Analysis	Health Quality Ontario	May 2016
Vertebroplasty and kyphoplasty for the treatment of vertebral compression fractures: an evidenced‐based review of the literature	McGirt	June 2009
Percutaneous techniques in the treatment of spine tumors: what are the diagnostic and therapeutic indications and outcomes?	Mendel	October 2009
**Disc replacement**		
Clinical outcomes of total disc replacement in the cervical spine		
Artificial total disc replacement versus fusion for the cervical spine: a systematic review	Zechmeister	February 2011
Clinical Outcomes of Treating Cervical Adjacent Segment Disease by Anterior Cervical Discectomy and Fusion Versus Total Disc Replacement: A Systematic Review and Meta‐Analysis	Lu	August 2019
Total disc replacement in the cervical spine: a systematic review evaluating long‐term safety	Anderson	February 2012
Comparison of Multilevel Cervical Disc Replacement and Multilevel Anterior Discectomy and Fusion: A Systematic Review of Biomechanical and Clinical Evidence	Li	August 2018
Systematic review and meta‐analysis of randomized controlled trials: comparison of total disk replacement with anterior cervical decompression and fusion	Yu	October 2011
Comparisons of Safety and Clinical Outcomes Between Multiple‐level and Single‐level Cervical Disk Replacement for Cervical Spondylosis: A Systematic Review and Meta‐analysis	Jiang	December 2016
What is the superior surgical strategy for bi‐level cervical spondylosis‐anterior cervical disc replacement or anterior cervical decompression and fusion?: A meta‐analysis from 11 studies	Zhao	March 2018
Factors that may affect outcome in cervical artificial disc replacement: a systematic review	Kang	September 2015
A systematic review of the radiological outcomes of total disc replacement surgery		
Radiological follow‐up after implanting cervical disc prosthesis in anterior discectomy: a systematic review	Yang	September 2018
Clinical outcomes of total disc replacement in patients with degenerative disc disease		
Total disc replacement surgery for symptomatic degenerative lumbar disc disease: a systematic review of the literature	van den Eerenbeemt	August 2010
Total disc replacement for chronic discogenic low back pain: a Cochrane review	Jacobs	January 2013
Total disc replacement for chronic back pain in the presence of disc degeneration	Jacobs	September 2012
Total disc replacement in the lumbar spine: a systematic review of the literature	Freeman	August 2006
Artificial Total Disc Replacement Versus Fusion for Lumbar Degenerative Disc Disease: An Update Systematic Review and Meta‐Analysis	Li	December 2019
Mid‐ to long‐term results of total disc replacement for lumbar degenerative disc disease: a systematic review	Cui	December 2018
Cervical Artificial Disc Replacement Versus Fusion for Cervical Degenerative Disc Disease: A Health Technology Assessment	Health Quality Ontario	February 2019
The short‐term efficacy and safety of artificial total disc replacement for selected patients with lumbar degenerative disc disease compared with anterior lumbar interbody fusion: A systematic review and meta‐analysis	Mu	December 2018
Lumbar artificial intervertebral disc replacement: a systematic review	Thavaneswaran	March 2014
The safety and efficacy of hybrid surgery for multilevel cervical degenerative disc disease versus anterior cervical discectomy and fusion or cervical disc arthroplasty: a systematic review and meta‐analysis	Hollyer	February 2020
Efficacy and Safety of Total Disc Replacement Compared with Anterior Cervical Discectomy and Fusion in the Treatment of Cervical Disease: A Meta‐analysis	Cai	October 2020
Comparison of Multilevel Cervical Disc Replacement and Multilevel Anterior Discectomy and Fusion: A Systematic Review of Biomechanical and Clinical Evidence	Li	August 2018
Mid‐Term to Long‐Term Outcomes After Total Cervical Disk Arthroplasty Compared With Anterior Diskectomy and Fusion: A Systematic Review and Meta‐analysis of Randomized Controlled Trials	Byvaltsev	June 2020
